# Association Between Triglyceride/High-Density Lipoprotein Ratio and Premature Coronary Artery Disease in Young Saudi Population: A Case–Control Study

**DOI:** 10.3390/diagnostics16121922

**Published:** 2026-06-21

**Authors:** Thamir Al-khlaiwi, Ayman Alsaleh, Hessah Alshammari, Sara Abou Al-Saud, Manan Alhakbany, Abdulmalik Alqahtani, Aliah Alshanwani, Sarah Mazi, Muhammad Iqbal

**Affiliations:** 1 Department of Physiology, College of Medicine, King Saud University, Riyadh 11461, Saudi Arabiaaalshanawani@ksu.edu.sa (A.A.); 2 Department of Cardiac Sciences, King Fahad Cardiac Center, College of Medicine, King Saud University Medical City, King Saud University, Riyadh 11362, Saudi Arabia876ani@gmail.com (A.A.); 3 Department of Cardiac Sciences, College of Medicine, King Saud University, Riyadh 11472, Saudi Arabiasaboualsaud@ksu.edu.sa (S.A.A.-S.);

**Keywords:** premature coronary artery disease, triglyceride-to-high-density lipoprotein cholesterol ratio, young Saudi population, sensitivity, specificity

## Abstract

**Background/Objectives**: Limited research has evaluated the association between the triglyceride-to-high-density lipoprotein (TG/HDL) ratio and premature coronary artery disease (PCAD), particularly in Saudi Arabia. Therefore, this study aimed to investigate the association of the TG/HDL ratio with PCAD and to assess its sensitivity and specificity in a young Saudi population. **Methods**: This comparative retrospective case–control study utilized data collected from patients’ electronic medical records at King Saud University Medical City (KSUMC) between 2015 and 2023. The vessel score and Gensini score were used to evaluate the severity of coronary occlusion. The study population was divided into two groups: (1) a healthy control group consisting of blood bank donors, selected to exclude individuals with chronic diseases such as metabolic disorders and hypertension, with no evidence of coronary artery disease and aged ≤50 years (as confirmed by a cardiologist to rule out cardiovascular disease); and (2) patients with PCAD, aged ≤51 years, who underwent selective coronary angiography using the standard hospital procedure (right femoral artery approach). Coronary angiographic images were evaluated using right and left oblique views with cranial and caudal angulations. **Results**: A total of 898 subjects were included in the study, comprising 440 healthy controls and 458 patients with PCAD. Higher HbA1c levels were significantly associated with PCAD (adjusted OR = 13.03, 95% CI [7.32, 23.18], *p* < 0.001). Importantly, the TG/HDL ratio, the primary biomarker of interest, remained significantly associated with PCAD after full adjustment. Each unit increase in the TG/HDL ratio was associated with more than a threefold increase in the odds of PCAD (adjusted OR = 3.39, 95% CI [2.22, 5.16], *p* < 0.001), independent of age, sex, BMI, HbA1c, smoking, and total cholesterol levels. Among females, the TG/HDL ratio demonstrated an area under the curve (AUC) of 0.796, with an optimal cut-off value of 0.91, yielding 77.8% sensitivity and 71.4% specificity. Among males, the TG/HDL ratio yielded an AUC of 0.786, with a higher optimal cut-off value of 1.09 providing 73.4% sensitivity and 65.4% specificity. **Conclusions**: Our study indicates that the TG/HDL ratio and HbA1c are significantly associated with PCAD in young Saudi male and female populations, demonstrating good sensitivity and specificity. Females exhibited a lower cut-off value than males. Smoking and elevated cholesterol levels were also identified as prominent risk factors. However, the TG/HDL ratio did not distinguish between moderate and severe coronary stenosis, as assessed by the Gensini score.

## 1. Introduction

Coronary artery disease (CAD), including premature coronary artery disease (PCAD), remains the leading cause of morbidity and mortality worldwide [1,2,3]. PCAD typically occurs in men before the age of 45 years and in women before the age of 55 years, although these age thresholds vary across studies [4]. The prevalence of PCAD is increasing and is expected to rise further by 2050, largely due to traditional risk factors such as increased body mass index (BMI) and elevated fasting plasma glucose (FPG) as well as non-traditional risk factors [5].

Early exposure to cardiovascular disease (CVD) risk factors in young individuals increases the likelihood of developing cardiovascular-related diseases, symptoms, and all-cause mortality later in life [6,7,8]. Because PCAD occurs at a relatively young age, affected individuals are often at a critical stage of their personal, family and professional lives. The sudden onset of disease in this population can compromise their health and ability to fulfil family responsibilities, thereby imposing a substantial socioeconomic burden on society and negatively affecting overall public health [9]. A systematic analysis from the Global Burden of Disease study demonstrated a significant worldwide increase in the prevalence of PCAD [9]. However, these rates vary according to socioeconomic status, sex, and geographic region [9]. The onset of PCAD during early adulthood may lead to long-term physical limitations and psychological distress, reducing individuals’ ability to contribute fully to their families, communities, and the economy [9]. Given that a large proportion of CVD cases in young populations are preventable, early identification of individuals at risk is essential for improving outcomes and reducing disease burden. However, studies investigating PCAD and its risk factors in the Saudi population remain limited. Several established risk factors have been associated with the development of PCAD, including smoking, diabetes mellitus, hypertension, a family history of CAD, dyslipidemia, and obesity [3,10].

Analysis of the blood biomarkers, including the lipid profile and glucose levels, is a highly effective and widely used diagnostic tool for assessing CVD, atherosclerosis, heart failure, and stroke [11]. Dyslipidaemia is characterized by elevated total cholesterol, increased low-density lipoprotein (LDL), reduced high-density lipoprotein (HDL), and elevated triglyceride levels [12]. An impaired lipid profile contributes significantly to the development of atherosclerosis, which is a primary cause of heart attack and stroke. The triglyceride-to-high-density lipoprotein cholesterol (TG/HDL) ratio has been recognized as a reliable indicator of cardiovascular risk in patients with cardiovascular disorders [13,14]. However, limited research has been conducted to evaluate its association with PCAD, particularly in Saudi Arabia. Therefore, this study aimed to investigate the association, sensitivity, and specificity of the TG/HDL-C ratio in relation to PCAD in a young Saudi population.

## 2. Methods

This study was conducted in the Department of Physiology, College of Medicine, King Saud University, Riyadh, Saudi Arabia. It is a comparative retrospective case–control study. Data were collected from patients’ electronic medical records at King Saud University Medical City (KSUMC) from 2015 to 2023. Sociodemographic and anthropometric data (age, gender, BMI, smoking status, nationality) were obtained.

The study population was divided into two groups: (1) a healthy control group consisting of blood bank donors, selected to minimize the presence of chronic diseases such as metabolic disorders and hypertension, with no evidence of coronary artery disease and aged ≤50 years (as evaluated by a cardiologist to rule out cardiovascular disease); and (2) patients with PCAD, aged ≤51 years, who underwent selective coronary angiography using the standard hospital procedure via the right femoral artery approach.

Inclusion criteria for the PCAD group included males and females aged ≤50 years with coronary artery disease confirmed by angiography. The exclusion criteria included patients with hematological disorders, variant angina, or chronic kidney disease. Institutional Review Board (IRB) approval was obtained from the College of Medicine, King Saud University (No. E-22-6747, dated 4 April 2022), in accordance with the Declaration of Helsinki. The privacy and confidentiality of all participants were maintained.

Clinical measurements and biochemical markers, including LDL, HDL, total cholesterol, triglycerides, glycated hemoglobin (HbA1c), and blood pressure, were obtained during coronary angiography. Hospital reference ranges were used to define normal and abnormal values (with cut-off points for risk factors presented in the tables). Participants were classified as Saudi or non-Saudi based on their nationality records.

For assessment of occlusion severity, the Vessel score and Gensini score were used. The Vessel score reflects involvement of the main coronary vessels: left anterior descending artery (LAD), left circumflex artery (LCx), and right coronary artery (RCA), with significant stenosis (>50%). Based on the Vessel score, patients were classified as having single-, double- or triple-vessel disease. Regarding occlusion severity, the Gensini score was used. It evaluates the location, extent, and degree of arterial occlusion, with a score of zero indicating no occlusion. Coronary imaging was assessed using right and left oblique views with cranial and caudal positions.

Any occlusion in at least one of the main coronary arteries, LM (left main), LAD, LCx, or RCA, was considered as an inclusion criterion for the CAD group (including patients with acute coronary syndrome and those undergoing planned diagnostic testing for suspected chronic coronary syndrome).

### Statistical Analysis

The mean and standard deviation were used to describe continuous measured variables, while frequencies and percentages were used for categorically measured variables. The Kolmogorov–Smirnov statistical normality test was used to assess the statistical normality assumption for metric variables. The chi-squared test of independence was used to assess correlations between categorical variables, and the independent samples *t*-test was applied to examine statistical mean differences in metric scores across levels of dichotomous variables. However, the Mann–Whitney U test was used in cases where the assumptions of parametric testing were violated, as an alternative to the independent samples *t*-test. The Area Under the Receiver Operating Curve (AUC-ROC) was used to evaluate the discriminative ability of the TG/HDL ratio for PCAD. Multivariable binary logistic regression (MBLR) was applied to identify significant predictors of PCAD in the sample, and the association between independent predictor variables and the outcome (dependent variable) was expressed as multivariable adjusted Odds Ratios with corresponding 95% confidence intervals.

The relevance of associated variables was determined based on an extensive literature review and preliminary bivariate analyses. All predictors considered relevant to PCAD were tested as sets in sequential iterative models aiming at parsimony and diagnostic accuracy of the model. The Youden index was used to determine the optimal cut-off value for the TG/HDL ratio. IBM SPSS Statistics Version 29 was used for statistical analysis, and the level of statistical significance was set at α = 0.05.

## 3. Results

### 3.1. Sociodemographic and Clinical Characteristics of Healthy and Premature Coronary Artery Disease (PCAD) Groups

Table 1 presents the sociodemographic and clinical characteristics of the study population (*N* = 898), including 440 healthy controls and 458 patients with PCAD. Regarding gender distribution, PCAD was markedly more prevalent among males. While females constituted nearly half of the healthy group (46.8%), they represented only 9.8% of PCAD cases, whereas males accounted for 90.2% of those diagnosed with PCAD (χ^2^(1) = 152.5, *p* < 0.001). Patients with PCAD were significantly older than healthy subjects, with a mean age of 43.5 years (SD = 5.55) compared with 37.93 years (SD = 8.00) in the healthy group (t(779.1) = 12.1, *p* < 0.001). Age-group analysis further demonstrated a strong gradient, as individuals aged 41–50 years constituted 74.5% of PCAD cases but only 40.7% of healthy participants, whereas younger age groups were disproportionately represented among the healthy (χ^2^(2) = 116.6, *p* < 0.001). Significant differences were also noted for body weight and adiposity. Individuals with PCAD had higher mean body weight (82.16 kg vs. 73.28 kg; t(824.9) = 8.88, *p* < 0.001) and higher mean BMI (29.46 vs. 26.38 kg/m^2^; t(749.5) = 9.80, *p* < 0.001) compared with healthy participants. In categorical analyses, obesity—particularly obesity class I and II—was substantially more prevalent among those with PCAD, while underweight and normal BMI categories were more common in the healthy group (χ^2^(4) = 114.93, *p* < 0.001). No significant difference was observed for height between groups (*p* = 0.493). Nationality was also significantly associated with PCAD status. Although Saudi nationals comprised the majority of both groups, non-Saudi participants represented a significantly larger proportion of the PCAD group (19.9%) compared with the healthy group (9.3%; χ^2^(1) = 19.92, *p* < 0.001). Strong associations were identified for cardiometabolic comorbidities. A history of diabetes mellitus was present in over half of PCAD patients (51.3%), compared with virtually none of the healthy group (0.2%; χ^2^(1) = 302.3, *p* < 0.001). Similarly, hypertension was observed exclusively among PCAD patients (42.8%), with no cases reported in the healthy group (χ^2^(1) = 240.9, *p* < 0.001). Finally, smoking status differed markedly by PCAD status. Current smoking was highly prevalent among PCAD patients (50.0%) but uncommon among healthy participants (6.4%), whereas never-smokers constituted the majority of the healthy group (93.2%) but less than half of PCAD cases (42.8%). These differences were statistically significant (χ^2^(2) = 259.97, *p* < 0.001).

### 3.2. Hemodynamic and Biochemical Characteristics of Control Group and Patients with Premature Coronary Artery Disease (PCAD)

Bivariate analyses, Table 2, were performed to compare hemodynamic and laboratory parameters between healthy participants and PCAD patients. Participants with PCAD demonstrated significantly higher blood pressure levels compared with healthy controls. Mean systolic blood pressure was substantially elevated in the PCAD group (134.51 mmHg, SD = 24.64) relative to the healthy group (121.30 mmHg, SD = 11.98; t(317.2) = 7.90, *p* < 0.001). Similarly, diastolic blood pressure was higher among PCAD patients (83.12 mmHg vs. 74.66 mmHg; t(319.8) = 7.42, *p* < 0.001). Mean arterial pressure was also modestly but significantly higher in the PCAD group (*p* = 0.032), reflecting a more adverse hemodynamic profile. Marked differences were observed in glycemic status. Mean HbA1c levels were substantially higher among participants with PCAD (7.29%, SD = 2.10) compared with the healthy group (5.38%, SD = 0.44; t(498.9) = 19.20, *p* < 0.001). Consistently, none of the healthy participants met the criterion for elevated HbA1c (≥6.5%), whereas 50.7% of PCAD patients fell within this range (χ^2^(1) = 300.5, *p* < 0.001). Lipid parameters further distinguished the two groups. Mean total cholesterol was significantly higher among PCAD patients (4.82 mmol/L, SD = 1.34) than healthy participants (4.51 mmol/L, SD = 0.77; t(737.1) = 4.26, *p* < 0.001), and elevated total cholesterol (≥5.2 mmol/L) was more prevalent in the PCAD group (40.0% vs. 15.2%; χ^2^(1) = 68.31, *p* < 0.001). Although mean LDL-C levels did not differ significantly between groups (*p* = 0.062), categorical analysis revealed that LDL-C > 2.85 mmol/L was significantly more common among PCAD patients (48.5%) compared with healthy individuals (32.7%; χ^2^(1) = 23.04, *p* < 0.001). Pronounced differences were observed in TG and HDL levels. Mean serum TG levels were nearly doubled in the PCAD group (2.01 mmol/L, SD = 1.43) compared with the healthy group (1.06 mmol/L, SD = 0.41; t(533.9) = 13.72, *p* < 0.001), and elevated TG (>1.7 mmol/L) were observed in 46.9% of PCAD patients versus 8.9% of healthy participants (χ^2^(1) = 160.42, *p* < 0.001). Conversely, HDL-C levels were significantly lower among PCAD patients (1.01 mmol/L, SD = 0.33) than among healthy subjects (1.38 mmol/L, SD = 0.51; t(746.1) = 12.73, *p* < 0.001). Low HDL-C was present in 60.3% of PCAD patients compared with 25.0% of healthy participants (χ^2^(1) = 113.86, *p* < 0.001). Most notably, the TG/HDL ratio, the primary biomarker of interest, showed a marked separation between groups. The mean TG/HDL ratio was significantly higher among PCAD patients (2.26, SD = 2.16) than among healthy individuals (0.86, SD = 0.50; t(501.1) = 13.60, *p* < 0.001). In categorical analyses, an elevated TG/HDL ratio was present in 28.8% of PCAD patients but in only 1.1% of healthy participants (χ^2^(1) = 133.04, *p* < 0.001).

### 3.3. Coronary Angiographic Characteristics and Occlusion Severity Among PCAD

Table 3 presents participants with angiographically confirmed PCAD (*n* = 458). The burden of coronary involvement was substantial despite the relatively young age of the cohort. Regarding the number of affected major coronary vessels, single-vessel disease was the most common presentation, observed in 38.5% of patients. However, a large proportion exhibited more extensive disease, with 27.0% demonstrating two-vessel involvement and 34.5% presenting with three-vessel disease, indicating a high prevalence of multivessel coronary pathology. The median Gensini score was 46 (IQR = 55.62), reflecting moderate-to-severe coronary disease burden. Consistently, the median coronary vessel score was 2 (IQR = 1), aligning with the high proportion of patients exhibiting multi-vessel involvement.

### 3.4. Gender Differences in Coronary Artery Involvement and Angiographic Severity Among PCAD Patients

Bivariate analyses were conducted to examine gender-based differences in angiographic severity among patients with angiographically confirmed coronary artery disease (*n*= 458), as shown in Table 4. Given the non-normal distribution of angiographic scores, comparisons of continuous severity indices were performed using non-parametric tests, while categorical vessel involvement was assessed using chi-square analyses. A statistically significant difference was observed in Gensini coronary artery disease scores between females and males. Male patients exhibited higher median Gensini scores (median = 48, IQR = 54) compared with female patients (median = 32, IQR = 70), indicating more severe coronary atherosclerotic burden among males (Z = 2.30, *p* = 0.023). In contrast, no significant gender difference was found for the coronary vessel score, with both females and males showing a median score of 2 (females: IQR = 1; males: IQR = 2; Z = 0.509, *p* = 0.611).

### 3.5. Multivariable Predictors of Premature Coronary Artery Disease

A multivariable binary logistic regression analysis was performed to identify independent association of variables with PCAD after simultaneous adjustment for key sociodemographic, anthropometric, metabolic, behavioral, and lipid-related variables (*N* = 898), as shown in Table 5 and Table S1. The model demonstrated several strong and statistically significant associations. Male sex emerged as an independent predictor of PCAD, with males exhibiting nearly a fourfold higher odds of disease compared with females (adjusted OR = 3.82, 95% CI [2.01, 7.23], *p* < 0.001). Age remained a significant predictor even within the restricted age range (<50 years), with each additional year associated with a 7.3% increase in the odds of PCAD (adjusted OR = 1.07, 95% CI [1.03, 1.12], *p* < 0.001). Measures of adiposity were independently associated with disease risk. Higher BMI was linked to increased odds of PCAD, such that each one-unit increase in BMI corresponded to an approximately 20% increase in risk (adjusted OR = 1.20, 95% CI [1.12, 1.28], *p* < 0.001). In addition, non-Saudi nationality was independently associated with more than a twofold increase in PCAD odds (adjusted OR = 2.45, 95% CI [1.28, 4.68], *p* = 0.007), suggesting potential differences related to socioeconomic, occupational, or healthcare-access factors. Among metabolic markers, glycemic dysregulation showed the strongest association with PCAD. Higher HbA1c levels were associated with a markedly elevated risk, with an adjusted odds ratio exceeding 13-fold (adjusted OR = 13.03, 95% CI [7.32, 23.18], *p* < 0.001), underscoring the dominant role of glycemia in PCAD. Importantly, the TG/HDL ratio, the primary biomarker of interest, showed a significant association with PCAD after full adjustment. Each unit increase in the TG/HDL ratio was associated with more than a threefold increase in the odds of PCAD (adjusted OR = 3.39, 95% CI [2.22, 5.16], *p* < 0.001), independent of age, sex, BMI, HbA1c, smoking status, nationality, and total cholesterol levels. Behavioral and lipid-related factors also retained independent significance. Individuals who reported smoking shisha and/or cigarettes had more than six-fold higher odds of PCAD compared with non-smokers (adjusted OR = 6.44, 95% CI [3.74, 11.08], *p* < 0.001). Additionally, elevated total cholesterol (≥5.2 mmol/L) was independently associated with increased disease odds (adjusted OR = 2.69, 95% CI [1.51, 4.81], *p* < 0.001).

**Figure 1:**
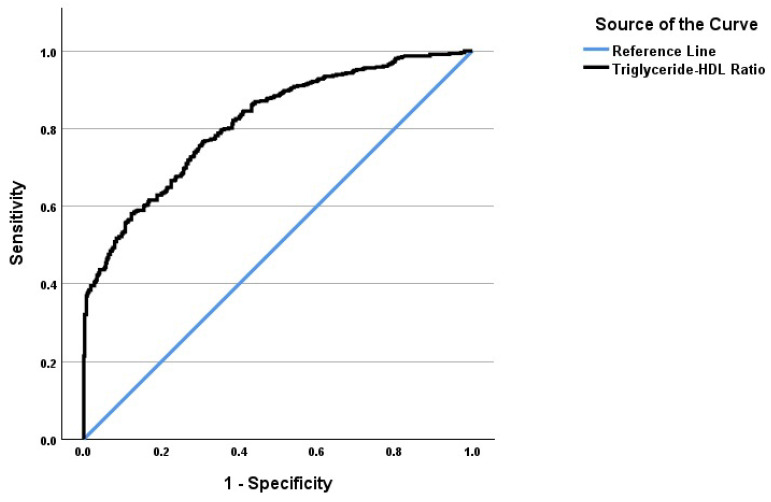
ROC curve of TG/HDL ratio predicting PCAD.

Table 6 and Figure 1 show that receiver operating characteristic (ROC) analysis was performed to evaluate the discriminative ability of the TG/HDL ratio for predicting PCAD among individuals aged 50 years or younger. In the overall sample, the TG/HDL ratio demonstrated significant discrimination between the groups, with an AUC of 0.816, 95% CI [0.789, 0.843], *p* < 0.001. The optimal threshold derived from the Youden index was 1.026, which achieved a sensitivity of 0.77 and specificity of 0.69 (Youden index = 0.46). Because metabolic parameters often differ between sexes, separate ROC analyses were conducted for males and females. Among females, the TG/HDL ratio produced an AUC of 0.796, and the optimal cut-off was 0.91, with 77.8% sensitivity, 71.4% specificity, and the highest gender-specific Youden index (0.491). For males, the TG/HDL ratio yielded an AUC of 0.786, and the corresponding optimal cut-off was higher at 1.09, providing 73.4% sensitivity, 65.4% specificity, and a Youden index of 0.388. These findings indicate that, although the TG/HDL ratio is strongly associated with the risk of PCAD across the entire cohort, sex-specific thresholds provide superior discriminative ability, with females showing a lower optimal cut-point and slightly stronger classification accuracy compared with males.

### 3.6. Discriminative Ability of Blood Biomarkers for Predicting Premature Coronary Artery Disease

**Figure 2:**
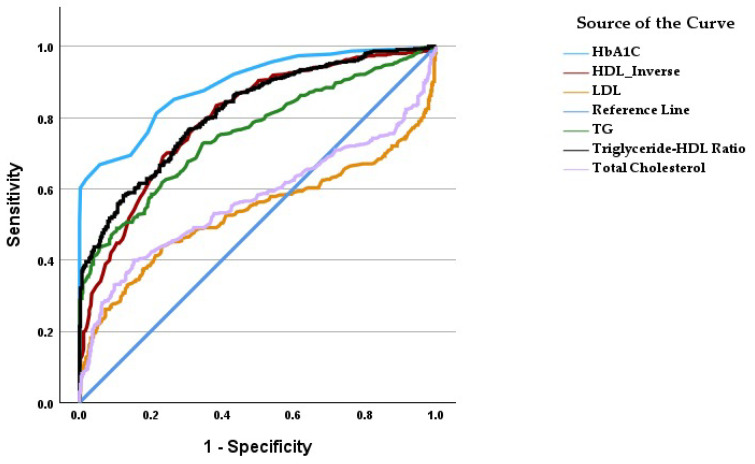
ROC curve analysis for various biomarkers predicting the PCAD among adults.

Receiver operating characteristic (ROC) curve analyses were conducted to compare the discriminative ability of multiple blood biomarkers in predicting PCAD (Table 7 and Figure 2). Statistically significant differences in predictive accuracy were observed across biomarkers, as reflected by their respective areas under the ROC curve (AUC). The serum glycated hemoglobin (HbA1c) demonstrated the highest discriminative ability, with an AUC of 0.891 (95% CI [0.870, 0.911], *p* < 0.001). This finding highlights the significant contribution of chronic glycemic dysregulation to the risk of PCAD. Importantly, the TG/HDL ratio showed very good discrimination, with an AUC of 0.816 (95% CI [0.789, 0.843], *p* < 0.001). The TG/HDL ratio outperformed traditional lipid parameters, including serum triglycerides alone (AUC = 0.755, 95% CI [0.723, 0.786], *p* < 0.001) and HDL (AUC = 0.796, 95% CI [0.767, 0.825], *p* < 0.001), highlighting its superior ability to integrate atherogenic and protective lipid components into a single risk marker. In contrast, total cholesterol demonstrated only poor discriminative ability (AUC = 0.568, 95% CI [0.530, 0.607], *p* < 0.001), while LDL showed no meaningful predictive value, with an AUC close to the chance level (0.532, 95% CI [0.493, 0.571]) and a non-significant *p*-value (*p* = 0.109). These findings suggest that conventional cholesterol metrics alone are limited in their ability to identify PCAD in younger adults. Visual inspection of the ROC curves further corroborated these results, with HbA1c and the TG/HDL ratio consistently demonstrating higher sensitivity across a wide range of specificities compared with other lipid markers.

### 3.7. Predictive Ability of TG/HDL Ratio for PCAD Severity

**Figure 3:**
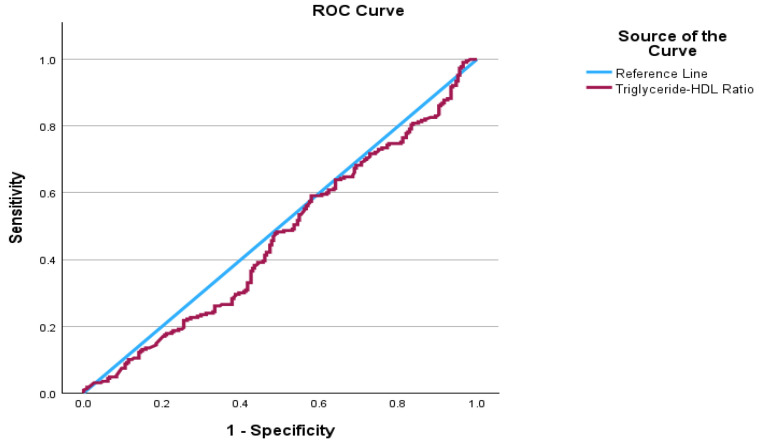
ROC curve: predictive ability of TG/HDL ratio for PCAD severity.

Additional ROC curve analyses were conducted to evaluate the ability of the TG/HDL ratio to discriminate severe coronary artery disease, as defined by elevated Gensini score thresholds (median value and a value of 39 points respectively) (Figure 3).

In contrast to its strong discriminative ability in predicting the presence of PCAD, the TG/HDL ratio demonstrated no significant discriminatory ability for identifying patients with more severe angiographic disease. The area under the ROC curve (AUC) for the TG/HDL ratio in predicting high Gensini score was equal to 0.464 (SE = 0.027), with a 95% confidence interval of 0.411 to 0.517. This value did not differ significantly from the null hypothesis of chance discrimination (AUC = 0.50; *p* = 0.184). Visual inspection of the ROC curve further confirmed the absence of meaningful separation between sensitivity and specificity, with the curve closely approximating the reference line. The analysis of the TG/HDL ratio predicting the Gensini based on a prior known value of 39 points also showed low predictive power, AUC = 0.483, *p*-value = 0.514. These findings indicate that, although the TG/HDL ratio is a strong marker for the presence of PCAD, it does not reliably distinguish between moderate and severe angiographic disease burden as quantified by the Gensini score.

## 4. Discussion

This study investigated the association between the TG/HDL ratio and PCAD in the Saudi population under 50 years of age and evaluated its discriminative capacity in terms of sensitivity and specificity. The TG/HDL ratio, along with HbA1c, was found to be highly sensitive and specific and remained independently associated with PCAD after adjusting for confounding factors in multivariable regression models. Females demonstrated a lower cut-off value than males. Further analysis indicated that the TG/HDL ratio did not distinguish between moderate and severe coronary artery occlusion when assessed using the Gensini score.

Despite significant advances in the diagnosis and prevention of CAD among older populations, data concerning younger individuals, particularly within the Saudi population, remain limited. The incidence of PCAD among individuals aged 35–54 years is increasing, particularly among women, with variations observed across different ethnic groups. In addition, the incidence of hospitalization for acute myocardial infarction among young patients has increased over a 20-year period [15]. The incidence of PCAD in Saudi Arabia is comparable to that reported globally and has been estimated to range from 5.5% to 30%, depending on the study settings [16]. Several risk factors, including smoking, diabetes mellitus, hypertension, a family history of CAD, dyslipidaemia, and overweight/obesity, contribute to the incidence of PCAD among young individuals both in Saudi Arabia and worldwide [3,17].

The TG/HDL ratio has been recognized as a reliable and novel biomarker associated with cardiovascular disease, metabolic syndrome, and insulin resistance. It is considered a valuable marker for assessing cardiovascular risk compared with individual lipid measurements such as LDL, HDL, or triglycerides alone [14]. In addition, the TG/HDL ratio has been shown to be associated with the coronary artery calcification score [13]. In our study, HbA1c and the TG/HDL ratio demonstrated strong association with PCAD individuals. Other lipid parameters, such as total cholesterol and LDL, showed comparatively lower discriminative ability. Because metabolic parameters differ between males and females, the data were analyzed separately for each sex. Among female patients, the Youden index identified a TG/HDL ratio cut-off value of 0.91 for predicting PCAD, with 77.8% sensitivity and 71.4% specificity. In contrast, the optimal TG/HDL ratio cut-off value for male patients was higher at 1.09, with 73.4% sensitivity and 65.4% specificity. Therefore, although the TG/HDL ratio demonstrated good sensitivity and specificity in both sexes, females exhibited a lower optimal cut-off value than males. TG/HDL ratio cut-off values vary among different populations [18,19]. A study by Singh et al. (2020) reported a cut-off value of 0.892 (95% CI) for predicting CAD risk in patients across different age groups (20–60 years) [20]. These findings further support the presence of sex-related differences in PCAD [21].

The TG/HDL ratio serves as an excellent marker for predicting adverse cardiovascular events in patients with acute coronary syndrome (ACS) undergoing percutaneous coronary intervention (PCI), and it correlates with coronary plaque characteristics, thereby helping to identify high-risk patients [21,22]. Furthermore, a study by Aydin et al. (2025) reported that the TG/HDL ratio showed a strong correlation with lesion burden, outperforming LDL-C, and may serve as a reliable biomarker for identifying coronary plaque burden in patients presenting with first-time ACS [19]. However, some studies have questioned the independent association of the TG/HDL ratio with PCAD, depending on study design and population characteristics, particularly with limited consideration of its long-term effects [23,24]. In our study, this index was observed more frequently in cases and may serve as a possible independent marker associated with PCAD after adjustment for age, sex, BMI, HbA1c, smoking status, nationality, and total cholesterol levels. However, the TG/HDL ratio did not reliably distinguish between moderate and severe angiographic disease as quantified by the Gensini score.

Modifiable risk factors such as impaired glycemic control, smoking, dyslipidaemia, and family history are commonly associated with acute myocardial infarction (AMI) in young individuals [17]. In our multivariable regression analysis, HbA1c showed a strong association with PCAD, with an adjusted odds ratio of approximately 13-fold. HbA1c is known to be associated with an increased risk of PCAD in several studies. Young individuals (≤35 years) have demonstrated higher levels of BMI, LDL cholesterol, and glucose compared with age- and sex-matched controls [25]. In a large follow-up study including 608,474 individuals, HbA1c > 6.0% was associated with an increased risk of CAD and mortality, particularly in men [26]. In another study, a positive correlation was reported between HbA1c levels and disease severity, including the number of diseased vessels and SYNTAX score, in non-diabetic patients [27]. Thus, elevated HbA1c is a well-established independent risk factor for PCAD in both diabetic and non-diabetic individuals [28]. A very important consideration when evaluating the ability of HbA1c is that the results should be interpreted with caution. The magnitude of the observed odds ratio may have been influenced by the study design, as virtually none of the control subjects had diabetes, whereas approximately half of the PCAD patients met the criteria for diabetes. This imbalance likely contributed to the very large effect estimate.

The TG/HDL-C ratio is associated with PCAD due to underlying insulin resistance, systemic inflammation, and an atherogenic lipid profile. The metabolic processes involved in insulin resistance include impaired lipid metabolism, reflected by abnormal triglyceride, HDL, and LDL levels [29]. During insulin resistance in adipose tissue, increased breakdown of stored fats elevates free fatty acid levels in the bloodstream. Consequently, the liver takes up these free fatty acids and converts them into triglycerides, packaging them into very-low-density lipoprotein (VLDL) particles, which results in elevated triglyceride levels. These VLDL particles are more susceptible to oxidation, glycation, and arterial uptake, thereby increasing the risk of coronary artery disease [30]. HDL particles may protect against cardiovascular disease through mechanisms such as reverse cholesterol transport from peripheral tissues and anti-inflammatory activities [31]. Hepatic lipase hydrolyzes triglycerides within HDL particles, producing smaller and less stable HDL particles. The activity of this enzyme is increased during insulin resistance, and these smaller HDL particles are rapidly cleared from the circulation [32]. Furthermore, insulin resistance promotes a pro-inflammatory state through multiple mechanisms. For example, low HDL levels and excessive circulating free fatty acids act as danger signals, triggering the release of pro-inflammatory cytokines [33]. Thus, the TG/HDL-C ratio is a key marker of insulin resistance and dyslipidemia and strongly correlates with PCAD progression through a combination of pathophysiological and metabolic mechanisms.

Tobacco use and smoking have emerged as major risk factors for AMI in young patients, with a prevalence of 70–90%, and smoking cessation has been shown to reduce the incidence of AMI by approximately 50% [17]. Smoking contributes to endothelial dysfunction, inflammation, and atherothrombotic processes [34]. Our data showed that smoking (shisha and/or cigarettes) was associated with more than six-fold higher odds of PCAD compared with non-smokers.

It is important to consider that, in this study, the Gensini score and coronary artery lesion score were used to confirm the diagnosis of primary coronary artery disease. The pathophysiology of CAD is complex and multifactorial, involving genetic, metabolic, lifestyle, and quality-of-life factors. Furthermore, PCAD frequently presents in multiple vessels with extensive and severe stenosis. Last but not least, the PCAD group included both patients presenting with acute coronary syndrome and those undergoing elective angiography for suspected chronic coronary syndrome. These subgroups differ in their clinical and metabolic characteristics, yet they were analyzed together. A separate subgroup analysis would be both valuable and informative, as it could provide a more detailed understanding of the associations observed in each clinical setting.

Limitations: Although the study has a relatively large sample size and employs a case–control design, it has several limitations. It lacks longitudinal follow-up to assess patient prognosis over time. In addition, as a retrospective study, it is subject to the inherent limitations of this design. Furthermore, being a single-center study, its generalizability may be limited and should be interpreted with caution. In addition, the use of a single lipid-related biomarker to characterize the complex and multifactorial nature of coronary artery disease severity should be interpreted with caution. Furthermore, although healthy control subjects underwent routine medical evaluation by the attending blood bank physician prior to donation, the presence of subclinical or undiagnosed cardiovascular disease cannot be completely excluded. One important concern that should be considered is that the control group contains virtually no individuals with diabetes or hypertension, whereas these conditions are highly prevalent in the PCAD group. Such a study design is likely to inflate both the observed odds ratios and the reported ROC performance. It would be valuable to compare PCAD patients without chronic diseases to a healthy control group, which could be considered in future studies.

## 5. Conclusions

Our study indicates that the TG/HDL ratio and HbA1c are significantly associated with PCAD in the young Saudi male and female population, demonstrating good sensitivity and specificity. Notably, females exhibited a lower cut-off value than males. Smoking and elevated cholesterol levels were also identified as significant risk factors. However, the TG/HDL ratio did not differentiate between moderate and severe coronary stenosis, as assessed by the Gensini score.

## Figures and Tables

**Table 1:** Sociodemographic and clinical characteristics of control group and patients with premature coronary artery disease (PCAD) (*N* = 898).

	Coronary Artery Disease			
	No, *n* = 440	Yes, *n* = 458	Test Statistic	*p*-Value
Sex				
Female	206 (46.8)	45 (9.8)	χ^2^(1) = 152.5	<0.001
Male	234 (53.2)	413 (90.2)		
Age (years), mean (SD)	37.93 (8.0)	43.5 (5.55)	t(779.1) = 12.1	<0.001
Age group				
18–30 years	77 (17.5)	14 (3.1)	χ^2^(2) = 116.6	<0.001
31–40 years	184 (41.8)	103 (22.5)		
41–50 years	179 (40.7)	341 (74.5)		
Body weight (Kg), mean (SD)	73.28 (12.28)	82.16 (17.35)	t(824.9) = 8.88	<0.001
Body Height (cm), mean (SD)	166.39 (9.15)	166.77 (7.76)	t(860.2) = 0.682	0.493
Body Mass Index (BMI), mean (SD)	26.38 (3.42)	29.46 (5.75)	t(749.5) = 9.80	<0.001
Body Mass Index (BMI) score				
Underweight	24 (5.5)	5 (1.1)	χ^2^(4) = 114.93	<0.001
Normal	104 (259)	91 (19.9)		
Over-weight	259 (58.9)	179 (39.1)		
Obesity class I	52 (11.8)	119 (26)		
Obesity class II	1 (0.2)	64 (14)		
Nationality				
Saudi	399 (90.7)	367 (80.1)	χ^2^(1) = 19.92	<0.001
Non-Saudi	41 (9.3)	91 (19.9)		
Medical History of Diabetes				
No	440 (100)	223 (48.7)	χ^2^ (1) = 302.3	<0.001
Yes	0 (0)	235 (51.3)		
Medical History of Hypertension				
No	440 (100)	262 (57.2)	χ^2^(1) = 240.9	<0.001
Yes	0	196 (42.8)		
Smoking Habit				
Never-smoker	410 (93.2)	196 (42.8)	χ^2^(2) = 259.97	<0.001
Ex-smoker	2 (0.5)	33 (7.2)		
Currently smoker	28 (6.4)	229 (50)		

χ^2^ = chi-squared test, t = *t*-test value. The numbers between brackets in the test statistic column are test degrees of freedom. *p* significance level was considered at 0.050.

**Table 2:** Hemodynamic and biochemical characteristics of control group and patients with premature coronary artery disease (PCAD) (N = 898).

	Coronary Artery Disease			
	No, *n* = 440	Yes, *n* = 458	Test Statistic	*p*-Value
Systolic Blood Pressure (mmHg), mean (SD)	121.3 (11.98)	134.51 (24.64)	t(317.2) = 7.9	<0.001
Diastolic Blood Pressure (mmHg), mean (SD)	74.66 (8.36)	83.12 (16.88)	t(319.8) = 7.42	<0.001
Mean Arterial Blood Pressure (MAP) (mmHg)			t(581.7) = 2.15	0.032
Serum Glycated Hemoglobin (HbA1C), mean (SD)	5.38 (0.44)	7.29 (2.1)	t(498.9) = 19.2	<0.001
**Serum HbA1c level**				
Low/normal < 6.5%	440 (100)	226 (49.3)	chi(1) = 300.5	<0.001
High ≥ 6.5%	0	232 (50.7)		
Serum Total Cholesterol (mmol/L), mean (SD)	4.51 (0.77)	4.82 (1.34)	t(737.14) = 4.26	<0.001
**Serum total cholesterol level**				
Normal < 5.2 mmol/L	373 (84.8)	275 (60)	chi(1) = 68.31	<0.001
High ≥ 5.2 mmol/L	67 (15.2)	183 (40)		
Serum Low Density Lipoprotein (LDL) (mmol/L), mean (SD)	2.64 (0.66)	2.76 (1.27)	t(694.9) = 1.87	0.062
**Serum LDL level**				
LDL ≤ 2.85, mmol/L	296 (67.3)	236 (51.5)	chi(1) = 23.04	<0.001
LDL > 2.85 mmol/L	144 (32.7)	222 (48.5)		
Serum Triglyceride (TG), (mmol/L), mean (SD)	1.056 (0.41)	2.01 (1.43)	t(533.9) = 13.72	<0.001
**Serum TG level**				
TG ≤ 1.7 mmol/L	401 (91.1)	243 (53.1)	chi(1) = 160.42	<0.001
TG > 1.7 mmol/L	39 (8.9)	215 (46.9)		
Serum High-Density Lipoprotein (HDL) Level (mmol/L), mean (SD)	1.38 (0.51)	1.012 (0.33)	t(746.1) = 12.73	<0.001
**Serum HDL level**				
Normal	330 (75)	182 (39.7)	chi(1) = 113.86	<0.001
Low	110 (25)	276 (60.3)		
TG/HDL Ratio, mean (SD)	0.86 (0.50)	2.26 (2.16)	t(501.1) = 13.6	<0.001
**TG/HDL ratio**				
Normal	435 (98.9)	326 (71.2)	chi(1) = 133.04	<0.001
High	5 (1.1)	132 (28.8)		

χ^2^ = chi-squared test, t = *t*-test value. The numbers between brackets in the test statistic column are test degrees of freedom. *p* significance level was considered at 0.050.

**Table 3:** Descriptive analysis of the coronary artery disease-diagnosed patients’ affected vessels and risk scores (*n*= 458).

Number of Affected Major Vessels	Frequency (%)
One	176 (38.5)
Two	124 (27)
Three	158 (34.5)
Gensini Coronary Artery Disease Score, median (IQR)	46 (55.62)
Coronary Vessel Score, median (IQR)	2 (1)

The number of affected vessels is presented as frequency (%). Gensini and vessel scores variables are presented as median (IQR).

**Table 4:** Descriptive bivariate analysis of gender differences in risk scores among PCAD patients (*n* = 458).

Severity Score	Female	Male	Test Statistic	*p*-Value
Gensini score, median (IQR)	32 (70)	48 (54)	Z = 2.3, df = 458	0.023
Vessel score, median (IQR)	2 (1)	2 (2)	Z = 0.509, df = 458	0.611

Gensini and vessel scores variables are presented as median (IQR).

**Table 5:** Multivariable logistic binary regression analysis of odds of PCAD patients. *N* = 898.

Variable	Multivariable Adjusted Odds Ratio	95.0% CI for OR		
		Lower Bound	Upper Bound	*p*-Value
Sex = Male	3.816	2.013	7.232	<0.001
Age (years)	1.073	1.032	1.116	<0.001
Mean Body Mass Index (BMI) Score	1.197	1.122	1.277	<0.001
Nationality = Non-Saudi	2.450	1.282	4.683	0.007
Mean Triglyceride–HDL Ratio Score	3.386	2.224	5.156	<0.001
Mean Serum Glycated Hemoglobin (HbA1C)	13.030	7.324	23.182	<0.001
Smoking Habit (current)	6.437	3.740	11.080	<0.001
High Serum Total Cholesterol (≥5.2 mmol/L)	2.693	1.509	4.805	<0.001
Constant	0.000			<0.001
**Dependent outcome: Diagnosis with PCAD (No/Yes).**				

Dependent outcome: Diagnosis with PCAD (No/Yes). Model overall significance: χ^2^(8) = 819.6, *p*-value < 0.001. Hosmer–Lemeshow Goodness-Of-Fitness, χ^2^(8) = 1.85, *p*-value = 0.985.

**Table 6:** Best-performing TG/HDL cut-offs according to Youden index metrics.

Parameters	Area Under ROC	TG/HDL Cut-Off	Sensitivity	Specificity	Youden Index
Overall	0.816	1.026	0.77	0.69	0.46
Female	0.796	0.91	0.778	0.714	0.491
Male	0.786	1.09	0.734	0.654	0.388

**Table 7:** Area under receiver operating characteristic curve of various blood biomarkers predicting PCAD patients.

Test Result Variable(s)		Asymptotic 95%CI AUC		
	Area Under ROC	Lower Bound	Upper Bound	*p*-Value
Triglyceride–HDL Ratio	0.816	0.789	0.843	<0.001
Serum Triglyceride Score	0.755	0.723	0.786	<0.001
Serum High-Density Lipoprotein (HDL)	0.796	0.767	0.825	<0.001
Serum Glycated Hemoglobin (HbA1C)	0.891	0.870	0.911	<0.001
Serum Total Cholesterol	0.568	0.530	0.607	<0.001
Serum Low-Density Lipoprotein (LDL)	0.532	0.493	0.571	0.109

## Data Availability

The original contributions presented in this study are included in the article. Further inquiries can be directed to the corresponding authors.

## Supplementary Materials

The following supporting information can be downloaded at: https://www.mdpi.com/article/10.3390/diagnostics16121922/s1, Table S1: Collinearity Statistics for Covariates Evaluated in the Multivariable Regression Analysis of BMI and the coronary artery disease.
