# Supplementary material for: Association Between Triglyceride/High-Density Lipoprotein Ratio and Premature Coronary Artery Disease in Young Saudi Population: A Case–Control Study
Source: Diagnostics (Basel). 2026 Jun 21;16(12):1922. doi: 10.3390/diagnostics16121922 (PMC13298638; doi:10.3390/diagnostics16121922)
Supplement: Supplementary file 1 [file diagnostics-16-01922-s001.zip › diagnostics-4334262-supplementary.pdf]

**Table S1.** Collinearity Statistics for Covariates Evaluated in the Multivariable Regression Analysis of BMI and the coronary artery disease.

| | Collinearity Statistics | |
|---|---|---|
| | **Tolerance** | **VIF** |
| Age-years | 0.847 | 1.180 |
| Nationality | 0.877 | 1.141 |
| Sex | 0.795 | 1.257 |
| TG/HDL ratio | 0.779 | 1.284 |
| HbA1C Score | 0.660 | 1.515 |
| smoking habit : Shisha+cigarrettes | 0.820 | 1.219 |
| LDL | 0.686 | 1.457 |
| Mean Arterial BP (mmhg) score | 0.854 | 1.171 |
| Coronary Artery Disease | 0.437 | 2.290 |
| Total cholesterol (>=5.2 mmol/L) | 0.603 | 1.659 |
